# Impact of Modified Transesophageal Echocardiography on Mortality and Stroke after Cardiac Surgery: A Large Cohort Study

**DOI:** 10.1155/2017/1857069

**Published:** 2017-09-11

**Authors:** Wouter W. Jansen Klomp, Carl G. M. Moons, Arno P. Nierich, George J. Brandon Bravo Bruinsma, Arnoud W. J. van't Hof, Jan G. Grandjean, Linda M. Peelen

**Affiliations:** ^1^Department of Cardiology, Isala, Dokter van Heesweg 2, 8025 AB Zwolle, Netherlands; ^2^Department of Clinical Epidemiology, Julius Center for Health Sciences and Primary Care, University Medical Center Utrecht, P.O. Box 85500, 3508 GA Utrecht, Netherlands; ^3^Department of (Thoracic) Anesthesia and Intensive Care, Isala, Dokter van Heesweg 2, 8025 AB Zwolle, Netherlands; ^4^Department of Cardiothoracic Surgery, Isala, Dokter van Heesweg 2, 8025 AB Zwolle, Netherlands; ^5^Department of Cardiology, Maastricht University Medical Center, P.O. Box 5800, 6202 AZ Maastricht, Netherlands; ^6^MIRA Institute for Biomedical Technology and Technical Medicine, University of Twente, P.O. Box 217, 7500 AE Enschede, Netherlands; ^7^Department of Anesthesiology, University Medical Center Utrecht, P.O. Box 85500, 3508 GA Utrecht, Netherlands

## Abstract

The aim of this study was to investigate the impact of perioperative screening with modified transesophageal echocardiography (A-View method). We compared, in consecutive patients who underwent cardiac surgery between 2006 and 2014, 30-day mortality and in-hospital stroke incidence, operated either with perioperative modified TEE screening (intervention group) or only with conventional TEE screening (control group). Of the 8,605 study patients, modified TEE was applied in 1,391 patients (16.2%). Patients in the intervention group were on average older (71 versus 68 years, *p* < 0.001) and more often females (31.0% versus 28.0%, *p* < 0.001) and had a higher predicted mortality (EuroSCORE I: 5.9% versus 4.0%, *p* < 0.001). The observed 30-day mortality was 2.2% and 2.5% in both groups, respectively, with multivariable and propensity-score adjusted relative risks (RRs) of 0.70 (95% CI: 0.50–1.00, *p* = 0.05) and 0.67 (95% CI: 0.45–0.98, *p* = 0.04). In-hospital stroke was 2.9% and 2.1% in both groups, respectively, with adjusted RRs of 1.03 (95% CI: 0.73–1.45) and 1.01 (95% CI: 0.71–1.43). In patients undergoing cardiac surgery, use of perioperative screening for aortic atherosclerosis with modified TEE was associated with lower postoperative mortality, but not stroke, as compared to patients operated on without such screening.

## 1. Introduction

In patients who undergo cardiac surgery, presence of aortic atherosclerosis is associated with an increased risk of postoperative stroke and mortality [1–4]. Atherogenic emboli released during aortic manipulation are thought to play a pivotal role in the pathophysiology of these complications, as these particles can cause cerebral ischemia. Other symptoms associated with these embolic complications are transient ischemic attack (TIA), convulsions, delirium, and postoperative cognitive decline [5, 6]. Screening for the presence of aortic atherosclerosis allows for a change in the surgical technique if aortic atherosclerosis is diagnosed, aimed at preventing the formation of such emboli. These changes include a different aortic cannulation site, choosing a different position for the aortic cross-clamp, the use of a dispersion cannula, cardiopulmonary bypass (CPB) through femoral or subclavian cannulation, or avoidance of CPB in a beating-heart “no touch aorta” procedure [7].

The distal ascending aorta (DAA) is most prone to the development of atherosclerosis [3]. Guidelines stress the importance of perioperative imaging of this part of the aorta with transesophageal echocardiography (TEE) or epiaortic ultrasound (EAU) [8–10]. Although epiaortic ultrasound is a highly accurate test, its use in practice is limited. On the contrary, TEE is widely used and has the advantage that it can be applied before sternotomy, yet visualization of the upper thoracic aorta is limited by the interposition of the air-filled trachea. Due to this so-called “blind-spot” the sensitivity of TEE for the diagnosis of atherosclerosis of the DAA is only 21% [11].

Echocardiographic visualization of these structures is possible using a modification to TEE, in which a balloon is introduced in the trachea. After inflation of the balloon with saline, the aortic arch and its branches can be accurately visualized through the trachea [12–14]. Previous studies have shown that modified TEE is safe to apply [12], the sensitivity for detection of atherosclerosis of the DAA was 95% (95% confidence interval [CI]: 92%–99%) [14], and its diagnostic accuracy was significantly better as compared to conventional TEE imaging [15].

Better diagnostic accuracy for modified TEE as compared to conventional TEE does not necessarily imply that it leads to improved care and consequently outcomes of patients [16, 17]. We therefore studied the impact of using modified TEE screening in patients undergoing cardiothoracic surgery, as compared to not using such screening, on the actual occurrence of postoperative mortality and stroke.

## 2. Methods

### 2.1. Study Design and Patients

**Figure 1:**
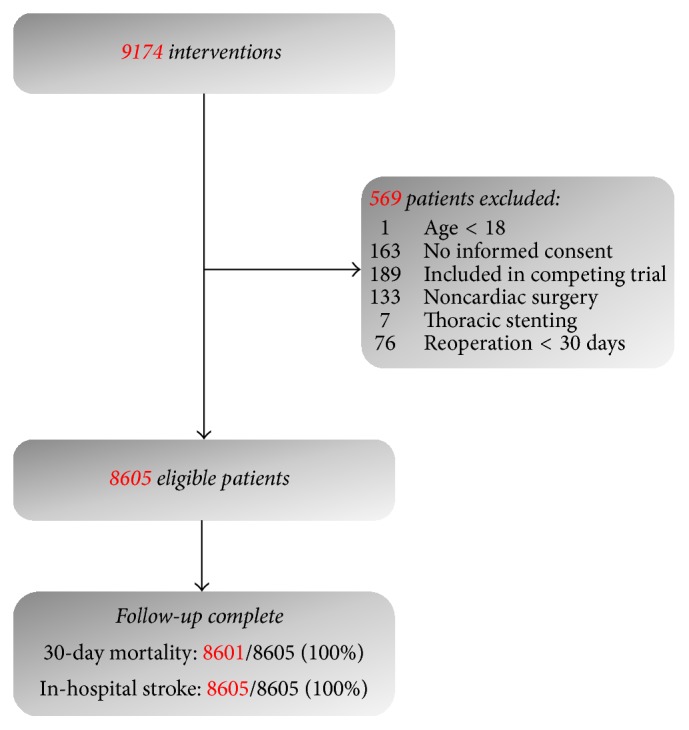
Flowchart of patient inclusion.

This observational large cohort study was conducted in the Isala Clinics, Zwolle, The Netherlands. All patients undergoing surgery that required a sternotomy between January 1, 2008, and December 31, 2014, were eligible. Excluded were patients aged below 18 (*N* = 1), patients who did not give informed consent (*N* = 163), those included in a competing study (*N* = 189), and patients who underwent noncardiac surgery (*N* = 133), thoracic stenting (*N* = 7), or reoperations within 30 days after the index surgery (Figure 1) [14]. Patients provided preoperative written informed consent for use of their data for clinical research, and the study conformed to the principals outlined in the Declaration of Helsinki. The institution's ethical committee waived formal evaluation of the study protocol.

#### 2.1.1. Interventions

**Figure 2:**
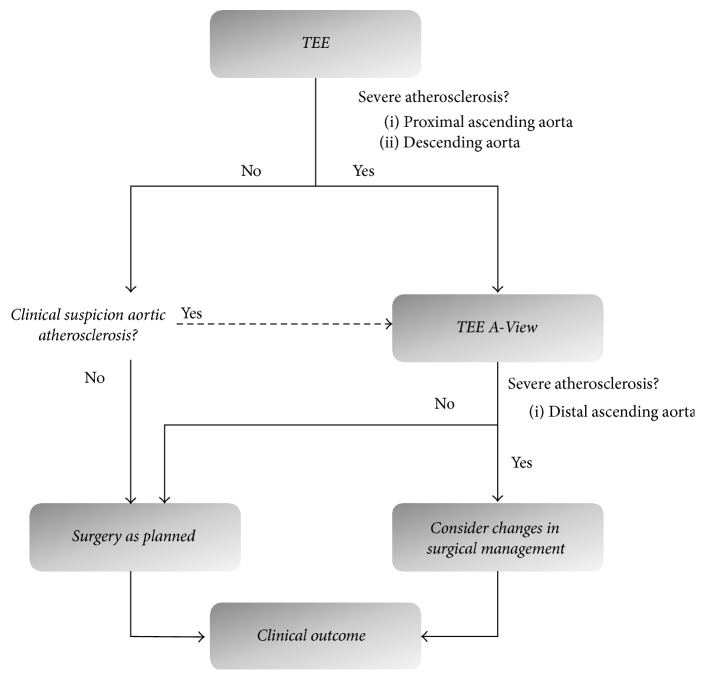
Flowchart of decision process to apply preoperative screening with modified TEE.

*Index Group*. Patients underwent TEE examination before sternotomy to screen for atherosclerosis of the proximal ascending aorta (PAA) and descending aorta. Severity of atherosclerosis was graded following Katz' classification (Table 1) [18]. In general, if TEE imaging showed grade three or higher atherosclerosis, modified TEE was added to visualize the DAA and aortic arch (Figure 2) [18]. This technique was described elaborately in previous publications [12–14]. In short, following a conventional TEE examination the “A‐View catheter” (Stroke2prevent, Zwolle, The Netherlands) was introduced via an endotracheal (ET) tube until the 24 cm marker lined up with a 24 cm marker on the ET‐tube. The correct position of the catheter in the left main bronchus was ascertained, and patients were preoxygenated. Then the balloon was inflated with 20–50 ml of sterile saline, after which imaging could be safely performed within two to three minutes. Three views of the aorta were acquired following a predefined protocol. First, starting from a conventional proximal ascending aortic transesophageal view, the DAA was visualized in a short-axis view by retracting the TEE probe to a depth of 25–30 cm of the incisors. Then, a long axis view was obtained by rotating the multiplane to 70–120 degrees. Finally, the aortic arch and its branch vessels were visualized by further retracting the probe in a 0-degree multiplane angle.

*Control Group*. The control group included surgical sternotomy patients operated on in the same hospital who did not undergo modified TEE. Perioperative screening with conventional TEE was used standardly in these patients; epiaortic ultrasound was used on indication.

#### 2.1.2. Endpoints

All-cause 30-day mortality was the endpoint of primary interest. Vital status was obtained through a patients' questionnaire that inquired about adverse events within 30 days after surgery. In patients who did not respond, the general practitioner was contacted. If the vital status was still unknown, the Dutch “Municipal Personal Records Database” was queried.

Secondary endpoint was in-hospital stroke, which was defined as a clinical suspicion of cerebral ischemia lasting longer than 24 hours. The diagnosis was confirmed by an attending neurologist who typically, although not mandatory, assessed presence of cerebral ischemia with cerebral computed tomography (CT) imaging. Stroke was recorded in the ongoing registry; to ascertain that all strokes were identified we also performed a chart review of all patients in whom a cerebral CT was performed during the index-hospitalization.

Finally, as an intermediate endpoint, modifications in the operative management of patients were assessed in both groups. Data was available for two surgical modifications: use of off-pump coronary artery bypass grafting (OPCAB) and cannulation with a different type of cannula (“Select 3D cannula,” Medtronic Inc., Minneapolis, MN) [19]. This cannula aims at preventing dislodgement of aortic atheroma by converting the outflow in three streams with a lower velocity [20].

#### 2.1.3. Potential Confounders

Due to our observational cohort design, the allocation to the use of modified TEE was clearly not random. Particularly, patients with a higher risk of having aortic atherosclerosis, based on the TEE results as well as on patient characteristics, were likely more indicated to undergo modified TEE screening during surgery. Since the same characteristics that indicate the use of modified TEE are also associated with a higher risk of postoperative stroke and mortality, the crude estimated effect association of the use of modified TEE (as compared to the control group) and the outcomes was likely distorted by these factors. We therefore considered the following confounders to be adjusted for in the analysis: logistic EuroSCORE, sex, age, hypertension, diabetes mellitus, unstable angina pectoris, previous myocardial infarction, previous percutaneous transluminal coronary angioplasty, previous cardiac surgery, preoperative creatinin, left ventricular ejection fraction (LVEF; <30%, 30–50%, and >50%), type of surgery (isolated coronary artery bypass grafting [CABG], isolated valve, CABG + valve, and others), and emergency indication. Age and EuroSCORE were modelled using a log transformation.

### 2.2. Statistical Analysis

#### 2.2.1. Unadjusted Analysis

First, the crude association of the use of modified TEE (intervention) as compared to not using this technique (control) was assessed for both outcomes using the relative risk (RR) and 95% CI.

#### 2.2.2. Multivariable Regression

Second, the association regarding the use of modified TEE as compared to the control group was corrected for the above-mentioned confounders, again for both endpoints. Rather than multivariable logistic regression, we used a binomial distribution with a log-link to obtain RRs with a 95% CI, which are more easily interpretable than odds ratios and are synchronous with the crude association measure [21, 22].

#### 2.2.3. Propensity-Score Analysis

Additionally, we corrected for confounding with propensity-score (PS) analysis [23]. Using logistic regression analysis a PS was created, with modified TEE as the dependent variable and the confounders as covariates. After checking that the range of the PS was similar for the exposed and the unexposed and that the score was well balanced over quintiles of the PS, the individual propensity scores were added to a model with only modified TEE status as a covariate.

Analyses were performed in SPSS 22.0 and R version 3.1.0. A *p* value < 0.05 was regarded statistically significant throughout this article.

## 3. Results

Of the 8,605 study patients, modified TEE was applied in 1,391 patients (16.2%).  Table 2 summarizes the baseline characteristics in comparison of both groups. The modified TEE (index) group included more females, and patients were on average older. These patients had more comorbid diseases, including hypertension, diabetes mellitus, extracardiac atherosclerosis, previous TIA or stroke, and COPD. Surgery in the index group more often included replacement of the aortic valve, ascending aorta, or (partial) aortic arch (Table 3). Duration of the extracorporeal circulation was similar in both groups, as was the postoperative ventilation time.

### 3.1. Change in Surgical Management

In the subgroup of patients who underwent coronary revascularization, the use of off-pump techniques was lower in the index group as compared to the control group (9.9% versus 15.7%; *p* < 0.001). Aortic cannulation with the “Select 3D cannula” was used more frequently in the modified TEE group than in the control group (2.5% versus 0.4%; *p* < 0.001).

### 3.2. Mortality

**Figure 3:**
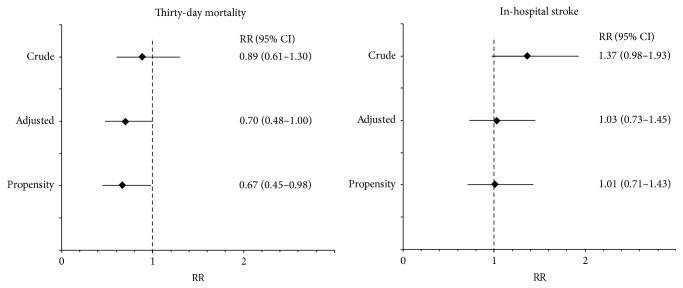
Crude, multivariable-adjusted and propensity-score adjusted relative risk of 30-day mortality and in-hospital stroke in patients with preoperative modified TEE screening compared to those without modified TEE.

The EuroSCORE-predicted mortality was 5.9% in the intervention group and 4.0% in the control group (*p* < 0.001). The observed 30-day mortality was 2.2% and 2.5% in both groups, respectively (RR: 0.89; 95% CI: 0.61–1.30, *p* = 0.55; Table 4; Figure 3). Perioperative screening for aortic atherosclerosis was associated with a lower mortality after both multivariate adjustment (RR 0.70, 95% CI: 0.48–1.00,* p* = 0.050) and propensity-score adjustment (RR 0.67, 95% CI: 0.45–0.98,* p* = 0.040).

### 3.3. Stroke

The predicted incidence of stroke was 4.1% (1.2%–4.8%) in patients with modified TEE and 2.8% (0.9%–3.4%) in the control group. The observed in-hospital stroke rate was 2.9% and 2.1%, respectively (RR 1.37; 95% CI: 0.98–1.93, *p* = 0.067; Table 4; Figure 3). The multivariable- and propensity-score adjusted RRs for in-hospital stroke were 1.03 (0.73–1.45) and 1.01 (0.71–1.43), indicating that stroke risk is similar in patients with and without modified TEE screening.

## 4. Discussion

This large nonrandomized intervention study showed that perioperative screening for aortic atherosclerosis with modified TEE was associated with a lower mortality after correction for confounding, as compared to patients without screening. The use of modified TEE was also associated with limited changes in the surgical management, but not with a lower incidence of stroke.

The evaluation of any new test should move beyond diagnostic accuracy, since an accurate test does not result in improved patient outcomes per se. First, a test should lead to an improved (or more timely) diagnosis; second it should lead to effective changes in patient management [16, 17, 24–27]. Considering modified TEE, we already showed that the diagnosis of aortic atherosclerosis was accurate and that the diagnosis was improved beyond conventional TEE imaging [14, 15]. Therefore, it was a logical next step to study the clinical effects of this test [7]. An ideal design would be a randomized diagnostic intervention study. However, randomized studies are expensive and time-consuming. Thus, we (first) performed a nonrandomized study to study the association between the use of modified TEE and clinical outcomes.

Modified TEE was used on indication, primarily if atherosclerosis of the proximal ascending aorta (PAA) or descending aorta was observed with conventional TEE. The baseline characteristics clearly reflect this “confounding by indication,” as patients with modified TEE screening were on average older and had more comorbid conditions than patients without modified TEE. Although the EuroSCORE-predicted mortality was higher in the former group (5.9% versus 4.1%, *p* < 0.001), the observed 30-day mortality was nonsignificantly lower (2.2% versus 2.5%, *p* = 0.55) and its use was associated with a lower propensity-score adjusted 30-day mortality (RR: 0.67; 95% CI: 0.45–0.98) as compared to the control group. Emboli related death is a common cause for postoperative mortality and may have contributed to this difference [28]. Another explanation for this difference may be that attending anesthesiologists differed not only in the use of modified TEE but also in other aspects of patient management. Since the effect of any diagnostic modality can only be operationalized through the actions of the observer, it would however be incorrect to adjust for the observers.

Both the expected and the observed incidence of stroke were higher in the modified TEE group; after correction for confounding, the stroke incidence was similar in both groups. This contrasted our expectation that modified TEE would be associated with a reduced incidence of stroke. Several explanations can be hypothesized for this finding. First, although we consider the large cohort a strength of this study, potentially we were still underpowered to show statistical significance of a difference in stroke incidence. Second, it is possible that there is still residual confounding because of unmeasured covariates. To conclude that use of modified TEE indeed reduces the risk of stroke, with similar incidences and a power of 0.80, would require an RCT with inclusion of 3825 patients per group.

Changes in patient outcome can only be achieved through improved patient management. We showed that the use of a so-called “3D dispersion cannula” was higher in the modified TEE group. This catheter is aimed at reducing the sandblasting effects of CPB on the posterior aortic wall by diverting the flow into multiple jets [19]. We also studied the use of off-pump revascularization, which is associated with a reduction in stroke especially in patients with aortic atherosclerosis [29]. Yet, in contrast to our expectations, an off-pump approach was used less often in the modified TEE group. Possibly, screening for aortic atherosclerosis was considered less needed in procedures without aortic manipulations.

It would have been of interest to have a more complete picture of other steps between test application and outcome such as subtle changes in the positioning of the aortic cross-clamp or CPB cannula. These were not collected in the registry and cannot be assessed retrospectively. Also, at the time of this study, we did not yet describe the indications for modified TEE in a protocol and the changes in patient management in the presence of aortic atherosclerosis were not standardized. Meanwhile, we have developed protocol providing a systematic approach for the diagnosis of atherosclerosis and the subsequent considerations in the intraoperative management [30].

Despite these limitations, the results of this hypothesis-generating study warrant further research to establish the association between modified TEE and patient outcomes [31]. A randomized diagnostic design should be considered to overcome the current limitations associated with “confounding by indication.” In such a study, the changes in the surgical management in the presence of aortic atherosclerosis should be further protocolized and registered in detail.

## Supplementary Material

Figure 1: Overview of TEE A-View technique. Schematic overview of the TEE A-View technique. By temporarily filling up the trachea with a saline filled balloon, the so called “blind spot” of conventional TEE is resolved. It enables a view of the upper mediastinum by physically looking through the trachea with ultrasound due to the lack of air in the trachea. After positioning of the A-View catheter in the trachea, the TEE probe is moved further into the esophagus in order to view the different images.
Figure 2: Clinical images of atherosclerosis with TEE A-View. (A) Upper Esophageal Distal Ascending Aorta Long Axis (LAX) A-View. On the posterior wall a mobile soft plaque is imaged, a high risk location if ECC is used in cardiac surgery or during Trans Aortic Valve Implantation procedures. (Image 2_A_). (B) Upper Esophageal Innominate Artery X-plane A-View. Direct through the trachea, the Innominate and left carotid artery are clearly visualized. This is of importance in case of atherosclerosis and aortic dissection. Direct flow from the aortic cannula into the innominate artery or left carotid artery during ECC might cause dislodgement of atherosclerotic debris with embolization into the brain. (Image 2B_1_ and Image 2B_2_). (C) Upper Esophageal Distal Ascending Aorta Color flow 3D and LAX A-View. Images of the so called “sand blasting” effect of CPB. These video's show the impact of different flow directions and patterns of aortic cannulae on their potential impact on plaque dislodgement from the distal ascending aorta, arch and its side branches. Imaging will guide the surgical team to choose the best option. (Image 2C_1_, Image 2C_2_ and Image 2C_3_).

## Figures and Tables

**Table 1:** Katz' classification of aortic atherosclerosis.

Grade atherosclerosis	Aspect	Clinical impact
1	Normal appearing aorta	Normal
2	Extensive intimal thickening	

3	Atheroma protruding < 5 mm	Diseased
4	Atheroma protruding > 5 mm	
5	Mobile atheroma	

**Table 2:** Baseline characteristics comparing patients with and without perioperative screening with modified TEE. BMI = body mass index, DM = diabetes mellitus, COPD = chronic obstructive pulmonary disease, TIA = transient ischemic attack, PTCA = percutaneous transluminal coronary angioplasty, and LVEF = left ventricular ejection fraction.

	Modified TEE (*N* = 1,391)	Nonmodified TEE (*N* = 7,214)	*p*
Age	71 (65–78)	68 (61–75)	<0.001
Female sex	431 (31.0)	2,023 (28.0)	0.026
BMI	27.0 (24.9–30.0)	27.0 (25.0–30.0)	0.38
History of			
Hypertension	867 (62.3)	3,490 (48.4)	<0.001
DM	365 (26.2)	1,629 (22.6)	0.003
Peripheral atherosclerosis	264 (19.0)	651 (9.0)	<0.001
TIA or stroke	192 (13.8)	545 (7.6)	<0.001
COPD	255 (18.4)	1015 (14.1)	<0.001
Myocardial Infarction	411 (29.5)	2,028 (28.1)	0.27
PTCA	242 (17.4)	1,064 (14.7)	0.012
Cardiothoracic surgery	109 (7.8)	405 (5.6)	0.001
Instable angina	80 (5.8)	350 (4.9)	0.16
LVEF			
>50%	870 (62.5)	4,725 (61.2)	0.069
30–50%	406 (29.2)	1,976 (31.3)	
<30%	115 (8.2)	508 (7.0)	
Preoperative creatinin	87 (75–103)	85 (73–100)	<0.001
Logistic EuroSCORE	5.9 (2.9–12.5)	4.0 (2.1–8.2)	<0.001

**Table 3:** Operative characteristics comparing patients with and without perioperative screening with modified TEE. ^*∗*^Percentage of patients with isolated coronary revascularization. CABG = coronary artery bypass grafting, CK = Creatinin Kinase, and CKMb = Creatinin Kinase MB isoenzyme.

	Modified TEE (*N* = 1,391)	Nonmodified TEE (*N* = 7,214)	*p*
Emergency indication	25 (1.8)	205 (2.8)	<0.001
Surgery			
CABG	942 (67.7)	5,033 (69.8)	0.13
Off-pump CABG	63 (9.9)^*∗*^	488 (15.7)^*∗*^	<0.001
Aortic valve surgery	481 (34.6)	1,860 (25.8)	<0.001
Mitral valve surgery	202 (14.5)	1,090 (15.1)	0.57
Aortic root replacement	36 (2.6)	164 (2.3)	0.48
Ascending aorta replacement	81 (5.8)	246 (3.4)	<0.001
(Partial) arch replacement	27 (1.9)	73 (1.0)	0.003
Extracorporeal circulation (min)	109 (75–164)	105 (78–153)	0.23
Blood loss during surgery (ml)	200 (150–300)	200 (200–350)	<0.001
Postoperative			
Ventilation	6 (4–9)	5 (4–8)	0.54
CK (max)	343 (206–692)	332 (210–598)	0.61
CKMb (max)	42 (28–68)	35 (25–63)	0.003

**Table 4:** Primary outcome measures comparing patients with and without perioperative screening with modified TEE.

	Modified TEE (*N* = 1,391)	Nonmodified TEE (*N* = 7,214)	*p*
Mortality, 30-day	31 (2.2)	177 (2.5)	0.55
Stroke, in-hospital	41 (2.9)	155 (2.1)	0.067

## Abbreviations

CABG: Coronary artery bypass grafting
CPB: Cardiopulmonary bypass
CT: Computed tomography
DAA: Distal ascending aorta
ET: Endotracheal
LVEF: Left ventricular ejection fraction
TEE: Transesophageal echocardiography
TIA: Transient ischemic attack
OPCAB: Off-pump cardiopulmonary bypass
PAA: Proximal ascending aorta.
