# Supplementary figures and images for: Impact of Modified Transesophageal Echocardiography on Mortality and Stroke after Cardiac Surgery: A Large Cohort Study
Source: Int J Vasc Med. 2017 Sep 11;2017:1857069. doi: 10.1155/2017/1857069 (PMC5611872; doi:10.1155/2017/1857069)

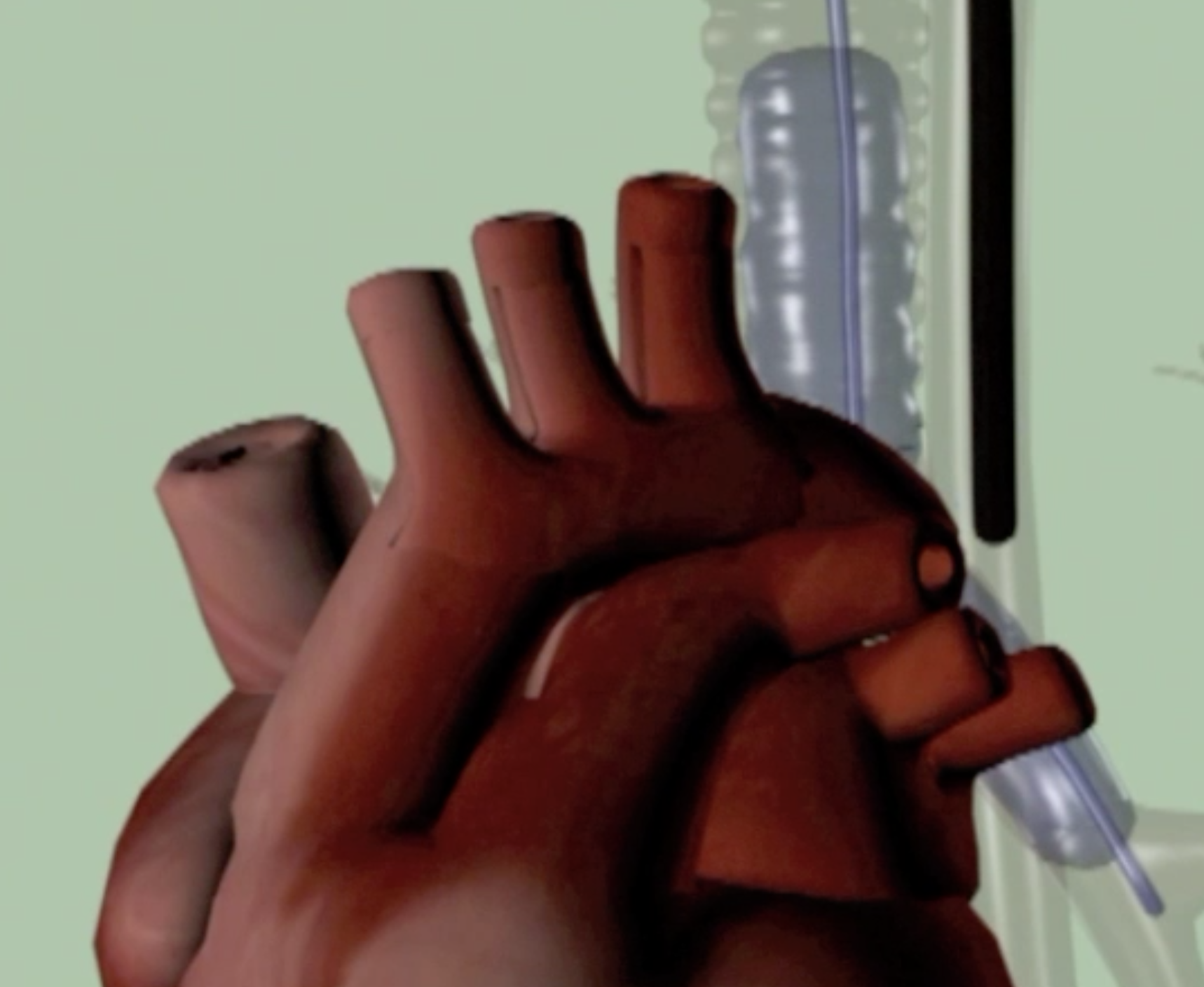

Supplement: Supplementary file 1 — Figure 1: Overview of TEE A-View technique. Schematic overview of the TEE A-View technique. By temporarily filling up the trachea with a saline filled balloon, the so called “blind spot” of conventional TEE is resolved. It enables a view of the upper mediastinum by physically looking through the trachea with ultrasound due to the lack of air in the trachea. After positioning of the A-View catheter in the trachea, the TEE probe is moved further into the esophagus in order to view the different images. Figure 2: Clinical images of atherosclerosis with TEE A-View. (A) Upper Esophageal Distal Ascending Aorta Long Axis (LAX) A-View. On the posterior wall a mobile soft plaque is imaged, a high risk location if ECC is used in cardiac surgery or during Trans Aortic Valve Implantation procedures. (Image 2A). (B) Upper Esophageal Innominate Artery X-plane A-View. Direct through the trachea, the Innominate and left carotid artery are clearly visualized. This is of importance in case of atherosclerosis and aortic dissection. Direct flow from the aortic cannula into the innominate artery or left carotid artery during ECC might cause dislodgement of atherosclerotic debris with embolization into the brain. (Image 2B1 and Image 2B2). (C) Upper Esophageal Distal Ascending Aorta Color flow 3D and LAX A-View. Images of the so called “sand blasting” effect of CPB. These video's show the impact of different flow directions and patterns of aortic cannulae on their potential impact on plaque dislodgement from the distal ascending aorta, arch and its side branches. Imaging will guide the surgical team to choose the best option. (Image 2C1, Image 2C2 and Image 2C3). [file 1857069.f1.zip › Fig 1_TEE A-View schematic.png]
